# Identification and Expression Analysis of Stress-Associated Proteins (SAPs) Containing A20/AN1 Zinc Finger in Cucumber

**DOI:** 10.3390/plants9030400

**Published:** 2020-03-24

**Authors:** Wei Lai, Yong Zhou, Rao Pan, Liting Liao, Juncheng He, Haoju Liu, Yingui Yang, Shiqiang Liu

**Affiliations:** 1 College of Bioscience and Bioengineering, Jiangxi Agricultural University, Nanchang 330045, China; 15797631915@163.com (W.L.); yongzhou@jxau.edu.cn (Y.Z.); hejc1994@163.com (J.H.); liuhaoju@mail.jxau.edu.cn (H.L.); 2 College of Agronomy, Jiangxi Agricultural University, Nanchang 330045, China; raopan0601@163.com (R.P.); llt18770156559@163.com (L.L.); yangyingui@163.com (Y.Y.); 3 Key Laboratory of Crop Physiology, Ecology and Genetic Breeding, Ministry of Education, Jiangxi Agricultural University, Nanchang 330045, China

**Keywords:** cucumber, stress-associated protein (SAP), abiotic stress, expression profile, gene family

## Abstract

Stress-associated proteins (SAPs) are a class of zinc finger proteins that confer tolerance to a variety of abiotic and biotic stresses in diverse plant species. However, in cucumber (*Cucumis sativus* L.), very little is known about the roles of *SAP* gene family members in regulating plant growth, development, and stress responses. In this study, a total of 12 *SAP* genes (named as *CsSAP1-CsSAP12*) were identified in the cucumber genome, which were unevenly distributed on six chromosomes. Gene duplication analysis detected one tandem duplication and two segmental duplication events. Phylogenetic analysis of SAP proteins from cucumber and other plants suggested that they could be divided into seven groups (sub-families), and proteins in the same group generally had the same arrangement of AN1 (ZnF-AN1) and A20 (ZnF-A20) domains. Most of the *CsSAP* genes were intronless and harbored a number of stress- and hormone-responsive cis-elements in their promoter regions. Tissue expression analysis showed that the *CsSAP* genes had a broad spectrum of expression in different tissues, and some of them displayed remarkable alteration in expression during fruit development. RT-qPCR results indicated that all the selected *CsSAP* genes displayed transcriptional responses to cold, drought, and salt stresses. These results enable the first comprehensive description of the *SAP* gene family in cucumber and lay a solid foundation for future research on the biological functions of *CsSAP* genes.

## 1. Introduction

Various abiotic stresses, such as salt, light intensity, drought, and extreme temperatures (cold and heat), are important causes of great damage to global crops by affecting their growth and reducing their average yield [1,2]. In order to resist and adapt to various environmental stresses, plants have developed complex molecular mechanisms that regulate the transcriptional levels of a number of stress-related genes to further control the signaling networks involved in stress responses. In recent years, stress-associated proteins (SAPs), a kind of zinc finger proteins (ZFPs), have been widely recognized as key molecular factors participating in the mediation of plant development and stress responses [3,4].

In previous studies, SAP gene family composition has been well characterized in different plant species. A high diversity in gene numbers has been reported, which range from 14 members in *Arabidopsis thaliana* to 57 members in *Brassica napus* [5,6,7,8,9,10]. Previous reports have revealed that the classical SAP proteins comprise an N-terminal A20 (ZnF-A20) domain and/or a C-terminal AN1 (ZnF-AN1) domain, which are specific zinc finger domains highly conserved across species [1,8,10,11]. The A20 and AN1 domains were shown to take part in immune response, and can interact with each other for protein–protein interactions and are involved in the transcriptional regulation of stress- and pathogenesis-related genes [12,13,14,15]. Additionally, the A20 and AN1 domains of some SAPs were reported to have ubiquitin ligase activity [16,17,18]. Besides the two zinc finger domains, some SAP proteins were found to possess an extra C-terminal Cys2-His2 domain [3,8,19].

There has been increasing evidence showing that many *SAP* genes are key regulators of abiotic/biotic stress responses. For example, *OsiSAP1* (*OsSAP1*) is the first *SAP* gene discovered in rice, and overexpression of *OsSAP1* in tobacco and rice increased their resistance to a variety of abiotic stresses by regulating the transcriptional levels of endogenous stress-related genes [12,20,21]. Likewise, overexpression of another rice *SAP* gene (*OsiSAP8*) in tobacco and rice also resulted in tolerance to multiple stresses such as salt, drought, and cold [22]. Enhanced stress tolerance was also observed in transgenic plants overexpressing different *SAP* genes, such as *AlSAP* from *Aeluropus littoralis* [23,24], *LmSAP* from the halophyte *Lobularia maritima* [25,26], *AtSAP9* and *AtSAP13* from *Arabidopsis thaliana* [19,27], *TaSAP17-D* from *Triticum aestivum* [28], and *PtSAP13* from *Populus trichocarpa* [11]. In addition, *OsSAP1*-overexpressing transgenic tobacco plants also displayed an increase in disease resistance against virulent bacterial pathogens with the up-regulation of defense-responsive genes [29]. Recent studies carried out in *Solanum lycopersicum* L. (tomato) also demonstrated the involvement of SlSAPs in plant response upon different biotic stresses [30,31]. Liu et al. [30] showed that SlSAP4 positively contributes to tomato immunity against *B. cinerea* by modulating the jasmonate and ethylene signaling pathways, and SlSAP3 positively regulates the immunity against *Pseudomonas syringae* pv. *tomato* DC3000 through the salicylic acid (SA) signaling pathway. In addition, SAPs were also found to play vital roles in regulating multiple aspects of biological processes, such as cell elongation [32], cell expansion [33], and glandular trichome development [7].

As one of the most widely distributed vegetable crops in the world, cucumber is sensitive to various environmental stimuli, which may severely threaten the production and quality [34]. Although numerous stress-responsive family genes have been found to play crucial roles in different stress responses of cucumber, such as *WRKY* [35], *bZIP* [36], *CDPK* [37], and *NAC* [38], the roles of *SAP* gene family members in response to environmental stresses are still unclear. In this work, we carried out the identification of *SAP* gene family members in cucumber, and systematically analyzed their chromosomal location, gene duplication events, gene structure, evolutionary relationships, promoter regions, and tissue expression profiles. Additionally, the expression profiles of cucumber *SAP* genes under various stresses (cold, drought, and salt) were investigated by RT-qPCR. Our findings are expected to shed light on the functional roles of *SAP* genes in cucumber.

## 2. Results

### 2.1. Identification and Chromosomal Location of the SAP Family Genes in Cucumber

In total, 12 *SAP* genes were identified in cucumber genome, and were named as *CsSAP1*-*CsSAP12* based on their physical locations on the chromosomes (Table 1). The gDNA lengths of *CsSAP* genes ranged from 414 to 3217 bp and the CDS lengths were from 414 to 870 bp. These *CsSAP* genes encoded proteins with 137–289 amino acids (aa) in length, with MWs from 14.78 (CsSAP7) to 31.81 (CsSAP6) kDa (Table 1). The predicted pIs and GRAVY values of the CsSAP proteins ranged from 7.95 (CsSAP1) to 9.52 (CsSAP4), and –0.793 (CsSAP2) to –0.332 (CsSAP1), respectively, suggesting that all of them are basic and hydrophilic proteins. In addition, the Plant-mPLoc results showed that all 12 CsSAP proteins were predictably located in the nucleus (Table 1).

**Figure 1:**
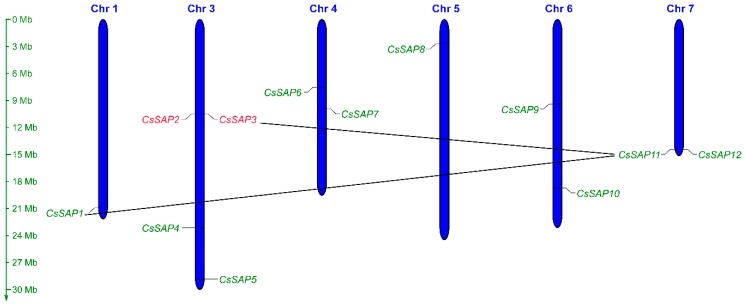
Distribution of the *CsSAP* genes among six cucumber chromosomes. The scale on the left represents the length of each chromosome displayed in megabase (Mb). Tandemly duplicated genes are colored with red, and segmentally duplicated genes are linked with black lines.

The 12 *CsSAP* genes were distributed on six chromosomes across the cucumber genome. Among them, chromosome 3 harbored the largest number of *CsSAP* genes (four genes), each of chromosomes 4, 6, and 7 harbored two *CsSAP* genes, and a single *CsSAP* gene was located in each of the chromosomes 1 and 5 (Figure 1). In addition, gene duplication analysis showed that *CsSAP2* and *CsSAP3* were tandemly duplicated genes, while two pairs of *CsSAP* genes, *CsSAP1/CsSAP11* and *CsSAP3/CsSAP11*, were identified as segmentally duplicated genes (Figure 1).

### 2.2. Phylogenetic Analysis of SAP Proteins in Cucumber and Different Plant Species

**Figure 2:**
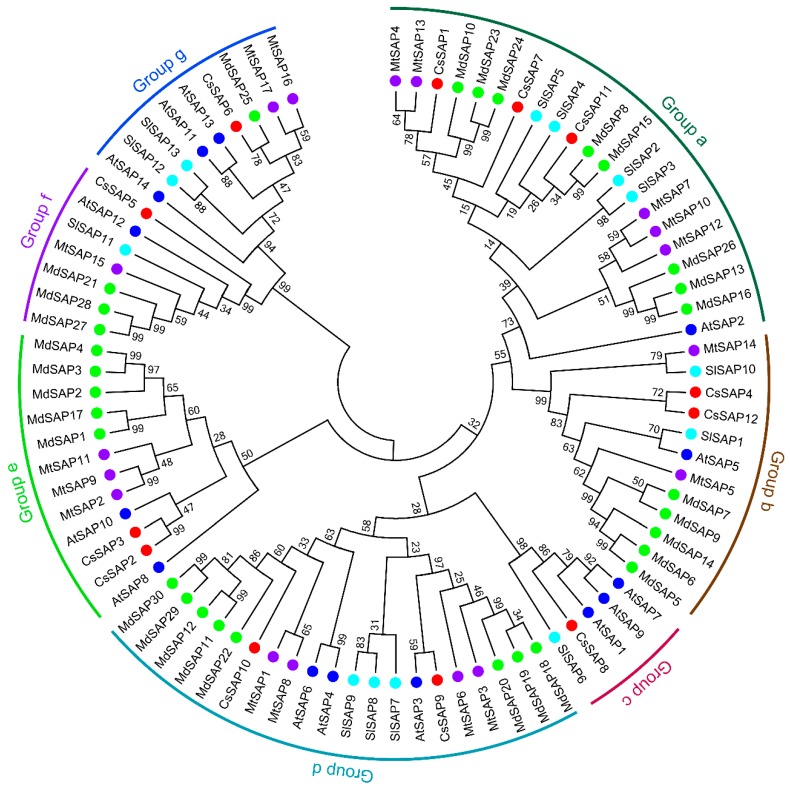
Phylogenetic analysis of stress-associated proteins (SAP) proteins from cucumber and four other plants. Sequence alignment was performed with full-length SAP amino acid sequences from different plant species using MAFFT with the default parameters, and the neighbor-joining (NJ) phylogenetic tree was created by MEGA 7.0 software using a bootstrap option of 1000 replications. Cs, *Cucumis sativus*; At, *Arabidopsis thaliana*; Sl, *Solanum lycopersicum*; Mt, *Medicago truncatula*; Md, *Malus domestica*. The accession numbers of SAPs for phylogenetic analysis are listed in Table S1.

To assess the evolutionary history of the SAP proteins, a phylogenetic tree was created with MEGA 7.0 using the SAP translated protein sequences from cucumber and four other plant species, including *A. thaliana* [10], *S. lycopersicum* [1], *M. truncatula* [3], and *M. domestica* [8]. As shown in Figure 2, these SAP proteins could be divided into seven groups (Group a–g). There were 21 members in Group a, which was the largest group. These members mainly possessed the AN1 and A20 domains, except for CsSAP7, MtSAP12, and MtSAP13, which only harbored one AN1 domain (Table 1) [3]. In addition, 12, 5, 20, and 12 members fell into groups b, c, d, and e, which also mainly possessed the AN1 and A20 domains. Group f included seven members, which all contained two AN1 domains. There were nine members in group g, which mainly possessed the AN1 and C2H2 domains, and the only exception was AtSAP14, which was lack of the C2H2 domain [10].

### 2.3. Conserved Motifs of SAP Proteins in Cucumber

**Figure 3:**
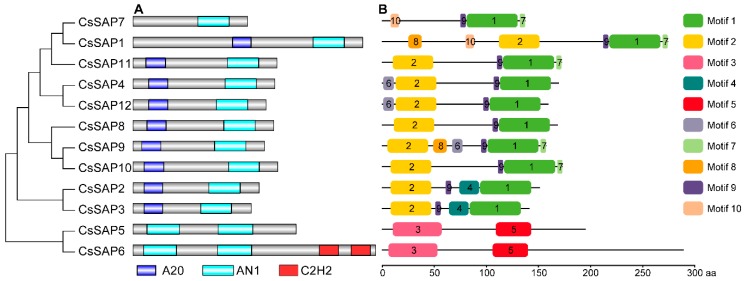
Conserved motif arrangement in cucumber SAP proteins according to the phylogenetic relationship. (**A**) Analysis of conserved domains of CsSAP proteins. (**B**) Conserved motif distributions of the CsSAP proteins annotated with the MEME server. Ten motifs are marked by different colors and their amino acid sequences are shown in Table S2.

The conserved domains of twelve putative CsSAP proteins were analyzed by Pfam and SMART servers. The results indicated that nine CsSAP proteins possessed both an A20 and an AN1 domain, while other three CsSAP proteins (CsSAP5, CsSAP6, and CsSAP7) contained AN1 domains but no A20 domains (Figure 3A). CsSAP7 contained only a single AN1 domain, while CsSAP5 and CsSAP6 possessed two AN1 domains. Besides two AN1 domains, CsSAP6 harbored two C2H2 domains, which was also observed in some SAP proteins from other plant species, such as SlSAP12 [1], MdSAP25 [8], GhSAP11A and GhSAP11D [9].

The MEME tool was employed to analyze the conserved motifs in the CsSAP proteins, and the motif distributions of these CsSAP proteins were obtained (Figure 3B; Table S2). Motif 2 made up the A20 domain, while motif 1, motif 3, and motif 5 were independently annotated as the AN1 domain (Table S2). Almost all CsSAP proteins had motif 9, except for CsSAP5 and CsSAP6. Motifs 4, 6, 7, 8, and 10 were present in 2, 3, 5, 2, and 2 CsSAP proteins, respectively (Figure 3B). Some evolutionarily well conserved CsSAP proteins harbored unique motifs. For instance, motif 4 was present in CsSAP2 and CsSAP3, while motif 10 was present in CsSAP1 and CsSAP7 (Figure 3B). In addition, besides motif 3 and motif 5, CsSAP5 and CsSAP6 had no additional motifs (Figure 3B).

### 2.4. Gene Structure of the CsSAP Genes

**Figure 4:**
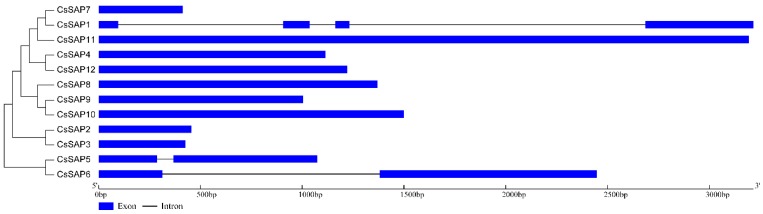
Compositions of introns and exons of *CsSAP* genes based on the phylogenetic relationship.

To investigate the structures of the *CsSAP* genes, the number and position of introns were predicted by comparing the CDS sequence and respective genomic DNA sequence using the GSDS tool (Figure 4). As a result, a majority of *CsSAP* genes (9 out of 12) were found to be intronless, two *CsSAP* genes (*CsSAP5* and *CsSAP6*) harbored one single intron, while *CsSAP1* contained three introns (Figure 4).

### 2.5. Promoter Region Analysis of the CsSAP Genes

**Figure 5:**
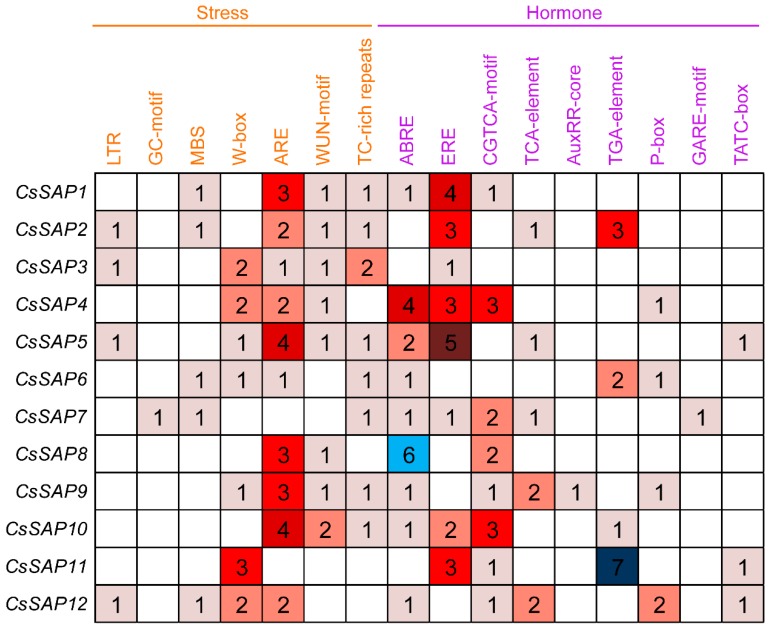
Types and numbers of stress- and hormone-responsive *cis*-elements in the promoter regions of *CsSAP* genes.

To study the potential roles of *CsSAP* genes, a search for *cis*-elements located at the promoter region of *CsSAP* genes and responsive to stress and hormones was carried out at the PlantCARE database. A total of seven and nine types of *cis*-elements associated with stress and hormone responses were identified, respectively (Figure 5). Among the stress-responsive *cis*-elements, ARE was the most abundant *cis*-element, with 10 out of the 12 *CsSAP* genes containing at least one ARE element. Specifically, the promoters of five *CsSAP* genes contained three or four ARE elements (Figure 5), indicating that they play possible roles in the anaerobic induction process. Additionally, nine kinds of hormone-responsive *cis*-elements were observed in *CsSAP* promoters, which were related to different hormones including ABA (ABRE), ethylene (ERE), MeJA (CGTCA-motif), salicylic acid (TCA-element), auxin (AuxRR-core and TGA-element), and gibberellin (P-box, GARE-motif and TATC-box) (Figure 5), suggesting that the *CsSAP* genes are associated with different hormone responses. In total, 22, 18, and 14 *cis*-elements were associated with ethylene, ABA, and MeJA responses, respectively, accounting for large proportions of the hormone-responsive *cis*-elements (Figure 5). Notably, seven TGA-elements, six ABREs, and five EREs were distributed in the promoter regions of *CsSAP11*, *CsSAP8*, and *CsSAP5*, respectively (Figure 5). These findings suggest that the *CsSAP* genes may play diverse roles in stress and hormone responses.

### 2.6. Expression Profiles of CsSAP Genes in Different Tissues and during Fruit Development

**Figure 6:**
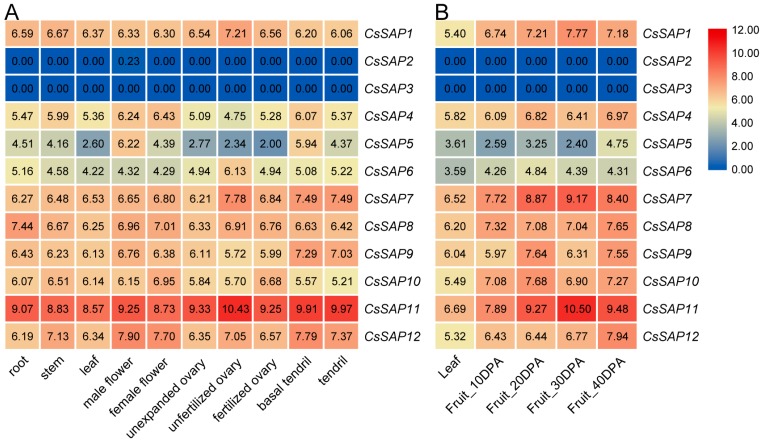
Expression levels of *CsSAP* genes in different cucumber tissues (**A**) and at different stages of fruit ripening (**B**). The expression levels were calculated as the log2-transformed RPKM+1 values based on data retrieved from available RNA-seq data. DPA, day after pollination.

The expression profile of *CsSAP* genes was examined based on the available RNA-seq data in different cucumber tissues and during fruit ripening [39,40]. As shown in Figure 6A, nearly all *CsSAP* genes were expressed in the tested tissues, except for *CsSAP2* and *CsSAP3*. Among these genes, *CsSAP1*, *CsSAP6*, *CsSAP7,* and *CsSAP11* displayed the highest transcriptional levels in unfertilized ovaries; *CsSAP4*, *CsSAP5*, *CsSAP10,* and *CsSAP12* exhibited much higher transcript abundance in flowers; while *CsSAP8* and *CsSAP9* showed the highest mRNA accumulation in roots and basal tendrils, respectively (Figure 6A). To further explore the possible roles of *CsSAP* genes in fruit development, the transcript levels of *CsSAP* genes at different fruit development stages were evaluated according to the previously reported RNA-seq data [40]. The transcriptome data showed that several *CsSAP* genes, such as *CsSAP1*, *CsSAP5*, *CsSAP7*, *CsSAP9*, *CsSAP11,* and *CsSAP12*, displayed remarkable transcriptions at certain time points during fruit development (Figure 6B). The tissue-specific expression patterns suggest that these genes may have different roles depending on the tissues.

### 2.7. Expression Patterns of CsSAP Genes under Abiotic Stress Treatments

**Figure 7:**
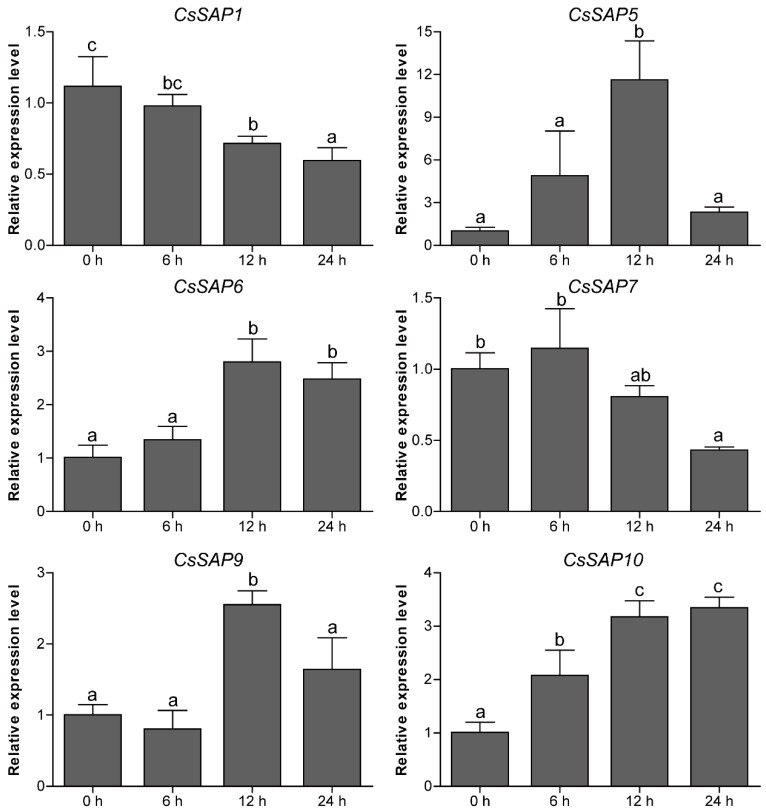
RT-qPCR analysis of six selected *CsSAP* genes under cold treatment. Error bars represent the standard error from three replicates, and different letters above the bars represent significant differences of the data at *P*  <  0.05 (Tukey’s test).

**Figure 8:**
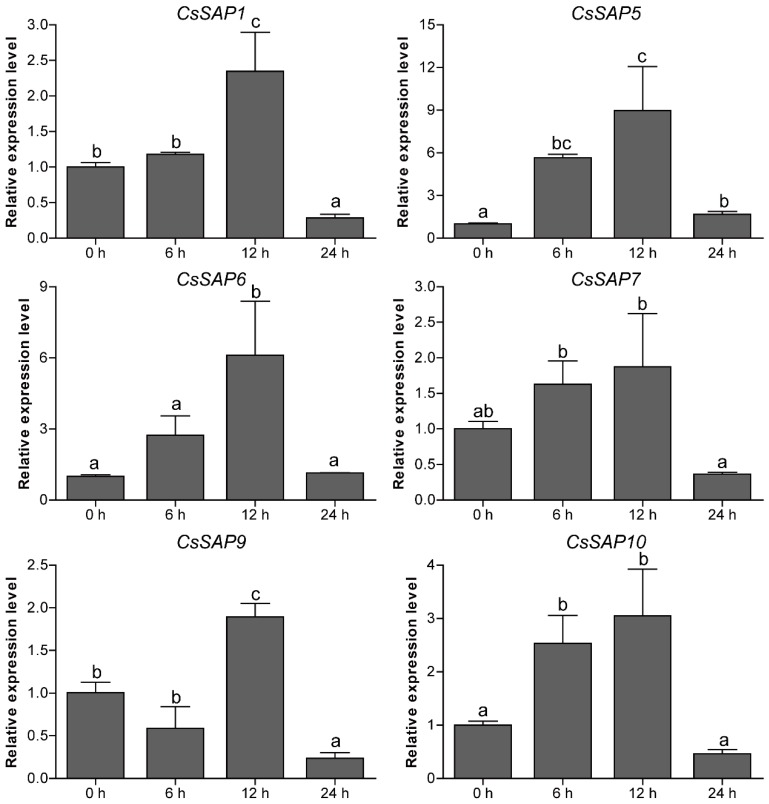
Expression profiles of six selected *CsSAP* genes under drought treatment as revealed by RT-qPCR. Error bars represent the standard error from three replicates, and different letters above the bars represent significant differences of the data at *P*  <  0.05 (Tukey’s test).

**Figure 9:**
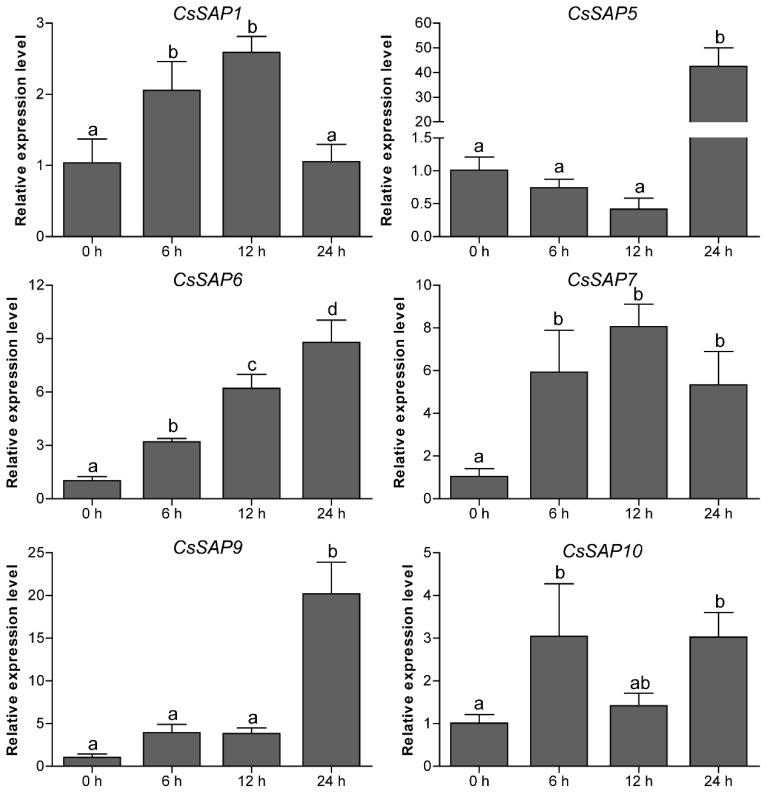
Expression profiles of six selected *CsSAP* genes under salt treatment as revealed by RT-qPCR. Error bars represent the standard error from three replicates, and different letters above the bars represent significant differences of the data at *P*  <  0.05 (Tukey’s test).

To unravel the roles of *CsSAP* genes in response to environmental stimuli, the expression profiles of six selected *CsSAP* genes under various abiotic stresses (cold, drought, and salt) were investigated by RT-qPCR. Under cold treatment, the transcript levels of *CsSAP5*, *CsSAP6*, *CsSAP9,* and *CsSAP10* were obviously increased at certain time points, particularly *CsSAP5*, while *CsSAP1* and *CsSAP7* were significantly inhibited (Figure 7). Under drought stress, all the *CsSAP* genes were up-regulated at 6 hps and/or 12 hps, followed by a further decrease at 24 hps (Figure 8). For salt stress, the transcription levels of all *CsSAP* genes were induced at specific time points or during certain periods (Figure 9). Notably, the transcription of *CsSAP6* was gradually induced during salt stress, and *CsSAP5* and *CsSAP9* were highly responsive to salt stress, with the transcript levels being up-regulated and peaking at a later time point (24 hps) (Figure 9). These results indicated that the *CsSAP* genes might have important effects when cucumber plants are exposed to different environmental stresses.

## 3. Discussion

In the present study, a total of 12 *SAP* gene members were identified and characterized in cucumber (Table 1). Genome-wide survey of *SAP* gene family composition has been performed in various plant species and there is a high variability in terms of the number of gene members. For example, 27 *SAP* genes were reported in *Glycine max* [5], 57 in *Brassica napus* [6], 16 in *Artemisia annua* [7], 30 in *Malus domestica* [8], 17 in *Medicago truncatula* [3], 37 in *Gossypium hirsutum* [9], 13 in *Solanum lycopersicum* [1], 18 in *Oryza sativa,* and 14 in *Arabidopsis thaliana* [10]. It turns out that there are fewer *SAP* family genes in cucumber than in these plants, possibly because of the tandem and segmental duplications of the *SAP* gene family. In a previous report, 36 out of the 37 *GhSAP* genes were found to form 18 pairs of putatively duplicated genes in cotton [9]. In apple, a total of 17 and two *MdSAP* genes have undergone tandem and segmental duplication events, respectively [8]. In the present study, only two segmental duplications and one tandem duplication were observed among the *SAP* genes in cucumber (Figure 1). In addition, a majority of CsSAP proteins (9 out of 12) harbor one A20 and one AN1 zinc finger domain, while the remaining three SAPs have no A20 domain (Table 1; Figure 3). However, SAP proteins with a single A20 domain were observed in some plants. For example, a total of seven BnaSAPs have a single A20 domain [6], and MdSAP22 and GmSAP23 also possess only one single A20 domain in apple and soybean, respectively [5,8].

Analysis of the evolutionary relationships of SAP proteins among cucumber and other four plants indicated that these SAPs can be divided into seven groups, and most proteins in the same group have the same arrangement of AN1 and A20 domains (Figure 2). We further examined the conserved motif distributions of cucumber SAP proteins according to the evolutionary relationship. A total of 10 conserved motifs were identified, and their distributions exhibited strong evolutionary conservation (Figure 3). Gene structure can also effectively reveal the evolutionary relationships among gene families. In this work, a large proportion of *CsSAP* genes (9 out of 12) were found to be intronless, which is in accordance with the previous reports [3,5,8,9]. The intronless genes were suggested to be able to reduce post-transcriptional processing for immediate responses to abiotic stresses [3]. These findings suggest that *SAP* genes are highly evolutionarily conserved in plants. Moreover, some *CsSAP* genes clustered together exhibited similar tissue expression patterns (Figure 2 and Figure 6). For example, *CsSAP1*, *CsSAP7,* and *CsSAP11* showed a board spectrum of expression in different tissues, and all displayed the highest expression in unfertilized ovaries (Figure 6A).

Previous reports have concluded that plant *SAP* genes play crucial roles in regulating a variety of abiotic and biotic stresses [22,30,31,41]. In the present study, a series of stress-responsive *cis*-elements were observed in the promoters of *CsSAP* genes (Figure 5), implying that the *CsSAP* genes may participate in stress responses. Additionally, RT-qPCR assay of the selected *CsSAP* genes under cold, drought, and salt stresses revealed that the transcript levels of these genes were obviously changed under the three stress conditions (Figure 7, Figure 8 and Figure 9). For instance, the transcript levels of *CsSAP5*, *CsSAP6*, *CsSAP9,* and *CsSAP10* were obviously elevated at certain time points under all of the three stresses, suggesting their potential roles in stress response. Both *OsiSAP1* and *OsiSAP8* are multiple stress-inducible genes in rice, and transgenic plants overexpressing either of them displayed tolerance to various abiotic stresses [12,20,21,22]. Similarly, *AtSAP5* was significantly up-regulated under salt, osmotic, drought, and cold stress conditions, and overexpression of *AtSAP5* conferred tolerance to a series of abiotic stresses in transgenic *Arabidopsis* plants [17,41]. Notably, *CsSAP5* displayed much more observably up-regulated expression than other *CsSAP* genes under the three stress conditions (Figure 7, Figure 8 and Figure 9), suggesting that it might contribute greatly to the resistance against stress in cucumber. It should be noted that *CsSAP1* and *CsSAP7* were suppressed by cold stress (Figure 7). They have closer evolutionary relationships than other *CsSAP* genes, and their encoded proteins display similar conserved motif arrangements (Figure 2 and Figure 3). These findings suggest that *CsSAP1* and *CsSAP7* may play negative roles in response to cold stress. However, no low-temperature-responsive (LTR) *cis*-elements were found in the promoter regions of *CsSAP1* and *CsSAP7* (Figure 5), implying that their promoters may harbor other unknown cold-responsive *cis*-elements. Some genes playing negative roles in stress tolerance were also observed in different plant species. For instance, ZFP185/OsSAP4 was found to have negative functions in stress tolerance and overexpression of *ZFP185* in rice increased its sensitivity to drought, cold, and salt stresses [42]. Down-regulation of *PagSAP1* in poplar resulted in significantly enhanced tolerance to salt stress with an increased K^+^/Na^+^ ratio in root and altered expression of genes associated with cellular ionic homeostasis [43]. However, the expression levels of *CsSAP1* and *CsSAP7* showed observable increases under drought and salt treatments at certain time points (Figure 8 and Figure 9). These findings indicate that the *CsSAP* genes may have vital regulatory functions in response to a variety of abiotic stresses, and cucumber may have developed diverse regulatory mechanisms when suffering from different environmental stimuli [2]. In a previous study, overexpression of *ZmAN13* in *Arabidopsis* plants led to enhanced tolerance to cold stress but increased sensitivity to salt and drought stress [15].

## 4. Materials and Methods

### 4.1. Identification of the SAP Family Members in Cucumber

The cucumber v2 protein sequences were acquired from Cucurbit Genomics Database (CuGenDB, http://cucurbitgenomics.org/). The A20 domain (PF01754) and AN1 domain (PF01428) were obtained from the Pfam server (http://pfam.xfam.org/), and employed for the identification of cucumber SAP members using HMMER3.0. In addition, the protein sequences of *SAP* family members of *Arabidopsis* and rice were retrieved from a previous report [10], and then used as queries to conduct an extensive BLASTp search against the cucumber v2 proteome. After removing the redundant sequences, each SAP candidate was checked via Pfam and SMART (http://smart.embl-heidelberg.de/) to verify that they had the A20 domain and/or the AN1 domain.

### 4.2. Protein Property and Sequence Analysis of Cucumber SAP Proteins

The ProtParam tool on the ExPASy server (http://web.expasy.org/protparam) was employed to predict the molecular weight (MW), isoelectric points (pI), and grand average of hydropathicity (GRAVY) of each cucumber putative SAP protein. The conserved motifs of cucumber SAP proteins were examined with MEME Version 5.1.0 (http://meme-suite.org/tools/meme) using the minimum and maximum width of the motif set to 6 and 50, and the number of different motifs was set to 10. The MEME results were illustrated with the TBtools software [44]. The distributions of A20, AN1, and C2H2 domains were visualized with the Illustrator for Biological Sequences (IBS) tool (http://ibs.biocuckoo.org/index.php). Subcellular localization analysis of each cucumber SAP protein was conducted with the Plant-mPLoc server (http://www.csbio.sjtu.edu.cn/bioinf/plant-multi/) [45]. For multiple sequence alignment, the full-length SAP amino acid sequences from cucumber and other plant species were aligned using the MAFFT server (https://www.ebi.ac.uk/Tools/msa/mafft/) with default settings. Subsequently, the alignment was imported into the MEGA 7.0 software to create a phylogenetic tree by the neighbor-joining (NJ) method with a bootstrap option of 1000 replications.

### 4.3. Chromosomal Distribution, Gene Structure, and Cis-Element Analyses of Cucumber SAP Genes

The information about chromosomal distribution, coding sequence (CDS), and genomic DNA (gDNA) sequence was retrieved from the cucumber genome database (http://cucurbitgenomics.org/organism/2). The chromosomal location of cucumber *SAP* genes was drawn with the MapInspect software following the procedure previously described [2]. For gene duplication analysis, the MCScanX software was employed to determine the tandem and segmental duplication events using the criteria from a previous study [46]. Gene structure was determined with the GSDS tool (http://gsds.cbi.pku.edu.cn/index.php) by comparison of CDS and respective gDNA sequence of each cucumber *SAP* gene. For identification of *cis*-elements located at the promoter regions of *SAP* genes, the 2.0-kb upstream sequence of the start codon (ATG) of each *CsSAP* gene was downloaded from the cucumber genome database. A search was performed by using the PlantCARE server (http://bioinformatics.psb.ugent.be/webtools/plantcare/html/).

### 4.4. Transcript Analysis of CsSAP Genes in Different Plant Tissues and during Fruit Ripening

To assess the tissue-specific expression profiles of *CsSAP* genes, the RNA-seq reads were downloaded in NCBI SRA database (https://www.ncbi.nlm.nih.gov/sra/?term=sra046916) and the expression profiles of *CsSAP* genes in flowers, leaves, ovaries, roots, stems, and tendrils were analyzed based on a previous study [47]. To gain insights into the possible roles of *CsSAP* genes during fruit development, their digital gene expression was derived from the fruitENCODE database (http://www.epigenome.cuhk.edu.hk/encode.html). All of the expression profiles of *CsSAP* genes were displayed as the reads per kilobase per million (RPKM) values, and the heat maps were illustrated with TBtools [44].

### 4.5. Transcript Analysis of CsSAP Genes under Different Abiotic Stresses

Cucumber (*Cucumis sativus* var. *sativus* line 9930) seedlings were grown in poly trays containing peat, sand, and pumice (1:1:1, v/v/v) within a growth chamber under a 16 h photoperiod, a night temperature of 18 °C and day temperature of 24 °C, and a relative humidity of 60–70%. Two-week-old cucumber seedlings were treated with different stress conditions including 4 °C (cold), NaCl (salt), and PEG (drought) as described in our previous reports [2,34]. Briefly, seedlings in the trays were transferred to 4 °C for cold stress. For salt and drought stress, 2-week-old seedlings were grown in liquid Murashige and Skoog (MS) medium containing 200 mM NaCl and 10% PEG-6000 (w/v), respectively. Samples were taken at 0, 6, 12, and 24 h after plant exposure to each stress condition. Each sample consisted of leaves taken from at least ten independent plants. Three biological repetitions were considered for each time point. All samples were frozen immediately in liquid nitrogen and stored at −80 °C until further processing.

Total RNA was isolated using the Eastep Super Total RNA Extraction Kit (Promega, Madison, WA, USA), and then about 3 μg RNA was reverse-transcribed into cDNA using the M-MLV reverse transcriptase (Invitrogen, Carlsbad, CA, USA) according to the manufacturers’ protocols as previously described [47]. The RT-qPCR was conducted with the Roche Lightcyler 480II PCR System using the TB Green Premix Ex TaqII Kit (TaKaRa, Dalian, China). All reactions were carried out with three technical replicates, and the RT-qPCR parameters were used as described previously [2]. *CsAct3* was selected as a housekeeping gene [2], and the 2^−ΔΔCt^ method was used to calculate the relative expression levels of *CsSAP* genes, which are shown relative to those at 0 h post stress exposure (hps). Gene-specific primers (Table S3) were designed by Primer Express software (Applied Biosystems, Foster City, CA, USA), and primer specificity was verified by Primer-BLAST at NCBI databases (https://www.ncbi.nlm.nih.gov/tools/primer-blast/). A melting curve analysis was conducted to ensure the specificity in amplification. All data were subjected to one-way analysis of variance (ANOVA) with Tukey’s test.

## 5. Conclusions

In the present study, we carried out the systematic identification and characterization of *SAP* gene family members in cucumber. Their characteristics, including genomic locations, gene duplications, evolutionary relationships, conserved domains and motifs, gene structures, and *cis*-elements in promoter regions, were analyzed based on bioinformatics methods. In addition, the expression patterns of *CsSAP* genes in different tissues and fruit development stages were investigated based on RNA-seq. Moreover, RT-qPCR assays showed that all six selected *CsSAP* genes were regulated by three stress treatments (cold, drought, and salt), suggesting that the *CsSAP* genes may play certain roles in stress responses. This study provides essential information for elucidating the potential roles of cucumber *SAP* genes in stress responses and may help to further promote the stress resistance of cucumber and other plants.

## Figures and Tables

**Table 1:** Identification and characterization of *SAP* family genes in cucumber.

Nomenclature	Locus	Chromosome	Chromosomal Position	gDNA (bp)	CDS (bp)	Protein					
						Length (aa)	MW (KDa)	pI	GRAVY	Subcellular Prediction	Zinc Fingers
*CsSAP1*	Csa1G613510	1	24072778–24075994	3217	825	274	30.38	7.95	−0.332	Nucleus	A20-AN1
*CsSAP2*	Csa3G177400	3	12052721–12053176	456	456	151	17.05	8.43	−0.793	Nucleus	A20-AN1
*CsSAP3*	Csa3G177900	3	12060993–12061418	426	426	141	15.68	8.21	−0.643	Nucleus	A20-AN1
*CsSAP4*	Csa3G697940	3	26515063–26516176	1114	510	169	18.72	9.52	−0.753	Nucleus	A20-AN1
*CsSAP5*	Csa3G829160	3	33158345–33159418	1074	588	195	21.2	8.8	−0.636	Nucleus	AN1-AN1
*CsSAP6*	Csa4G166950	4	8642222–8644669	2448	870	289	31.81	8.7	−0.541	Nucleus	AN1-AN1-C2H2-C2H2
*CsSAP7*	Csa4G290810	4	11340752–11341165	414	414	137	14.78	8.63	−0.46	Nucleus	AN1
*CsSAP8*	Csa5G157400	5	5586914–5588597	1684	507	168	18.14	8.47	−0.633	Nucleus	A20-AN1
*CsSAP9*	Csa6G152950	6	10845539–10846542	1004	474	157	17.83	8.49	−0.781	Nucleus	A20-AN1
*CsSAP10*	Csa6G452080	6	21537430–21538928	1499	522	173	18.58	8.3	−0.448	Nucleus	A20-AN1
*CsSAP11*	Csa7G428820	7	16527702–16530896	3195	519	172	18.59	8.89	−0.42	Nucleus	A20-AN1
*CsSAP12*	Csa7G428830	7	16533455–16534676	1222	480	159	17.8	9.4	−0.704	Nucleus	A20-AN1

## Supplementary Materials

The following are available online at https://www.mdpi.com/2223-7747/9/3/400/s1, Table S1: Accession numbers of SAPs for phylogenetic analysis. Table S2: Sequences and lengths of motifs among cucumber SAP proteins. Table S3: Gene-specific primers used for RT-qPCR.
