# Supplementary material for: Identification and Expression Analysis of Stress-Associated Proteins (SAPs) Containing A20/AN1 Zinc Finger in Cucumber
Source: Plants (Basel). 2020 Mar 24;9(3):400. doi: 10.3390/plants9030400 (PMC7154871; doi:10.3390/plants9030400)
Supplement: Supplementary file 1 [file plants-09-00400-s001.zip › Table S2.doc]

**Table S2.** Sequences and lengths of motifs among cucumber SAP proteins.

| **Motif** | **Conserved amino acid sequences** | **Width** |
| --- | --- | --- |
| 1 | CRKRVGLTGFKCRCGNLFCAVHRYPEKHNCSFDYKAAGREAIAKANPVVK | 50 |
| 2 | KPPEDPKLCVNNCGFFGSPATRNLCSKCYRDFLLKEEQAA | 40 |
| 3 | FPDLGKHCNFAECKQJDFLPFTCDCCQKVFCLEHRGFKRHDCPKADRW | 48 |
| 4 | LETCDHNDPAPPKTQNRCEI | 20 |
| 5 | CREJLTFSNTCKCKDCRLDVCLKHRFGPEHGCGGP | 35 |
| 6 | MAQRTEKEETE | 11 |
| 7 | EKLDKI | 6 |
| 8 | ALNDRLDWPQLLNP | 14 |
| 9 | PNRCSS | 6 |
| 10 | HKDFILKQD | 9 |
